# Designing a programme to train social workers on how to promote physical activity for disabled people: A Delphi study in the UK

**DOI:** 10.1111/hsc.13724

**Published:** 2022-01-17

**Authors:** Javier Monforte, Matthew Smith, Brett Smith

**Affiliations:** ^1^ 3057 Department of Sport and Exercise Sciences Durham University Durham England; ^2^ Institute of Health University of Cumbria Carlisle England

**Keywords:** curriculum development, Delphi method, health promotion, physical activity messaging, public health, social care, social work

## Abstract

Recently, social workers have been identified as a key messenger group for promoting physical activity (PA) to disabled people. Also identified is the need to train social workers in PA promotion. In response, the purpose of this article is to inform the design of a training programme prototype aiming to support social workers to become active PA messengers. We conducted a three‐round Delphi study to identify the essential contents and teaching methods for the programme, as well as the challenges that may jeopardise its success. Qualified experts on physical activity and health, social work, and/or disability working in the UK were invited to partake in the study. The response rates were 55% (33/60) in the first round, 79% (26/33) in the second and 77% (20/26) in the third rounds. Following the last questionnaire round, the experts reached consensus on 8 contents, 7 teaching methods and 10 challenges to success. The top three most important contents were: benefits of PA (1.05 ± 0.22), what PA means to disabled people (1.15 ± 0.36) and person‐centred PA planning (1.35 ± 0.57). The most relevant teaching methods were interactive activities and discussions (1.20 ± 0.51) and case studies (1.25 ± 0.43). Blended learning (1.85 ± 0.57) was preferred to e‐learning (2.20 ± 0.60) and face‐to‐face learning (2.10 ± 0.70). Lack of time (1.30 ± 0.46) and confidence (1.45 ± 0.59) were deemed vital challenges. However, consensus around other potential barriers such as lack of interest and commitment (1.30 ± 0.46), lack of buy in from employers (1.75 ± 0.70) and professional inertia (2.05 ± 0.67) suggest that a major challenge for long‐term impact is to convince key people that social work and PA promotion make a good match. The results of this study provide a valuable starting point evidence base for PA curriculum development. Future research will delve into expert opinions using in‐depth qualitative interviews. Participatory approaches including knowledge cafés will also be used to add more views of stakeholders with experiential knowledge.

**What is already known about the topic**

- The promotion of physical activity (PA) is largely entrusted to healthcare professionals.
- Disabled people have identified social workers as relevant PA messengers.
- In the UK, healthcare professionals are being trained in PA promotion, but social workers are not.

**What this study adds**

- Essential contents and teaching methods identified by qualified experts will help developing a training programme in PA promotion for social workers in the UK.
- A multiplicity of real and perceived barriers can undermine efforts to educate and train social workers in PA promotion.
- Embedding PA in the culture and identity of social workers will be key to achieve long‐term impact.

## 1 INTRODUCTION

Disabled people face a multitude of social, financial and physical barriers to being active, and remain one of the most physically inactive groups of society (Martin, 2013; Mascarinas & Blauwet, 2018). In England, they are twice as likely to be inactive when compared with non‐disabled people (Sport England, 2020), with inactivity at 43% among the former and 21% for the latter (Sport England, 2017). Estimates also suggest that just 18% of disabled adults engage in at least one physical activity (PA) session per week compared with 41% of non‐disabled adults (Sport England, 2015). Although there were steady increases in activity levels over the last years, drops have been registered as a consequence of the coronavirus pandemic and its associated restrictions (Sport England, 2021). Given this context, developing effective strategies to promote PA for disabled people has been designated an issue of national health importance (Jackson et al., 2021; Smith et al., 2018).

While it is unlikely to address inactivity on its own, PA messaging has been advanced as important for enhancing PA levels (Williamson et al., 2020). PA messaging refers to the process of designing, developing and delivering physical activity messages, which are communications containing some information and advice about PA. Central to PA messaging is the figure of the messenger: the person who knows and shares relevant PA messages. Within the specialised literature, three typical groups of PA messengers can be identified: community‐based organisations, peers with disabilities and healthcare professionals (HCPs), including doctors, nurses, occupational therapists and physiotherapists (Letts et al., 2011; Williams et al., 2018). Also highlighted in the literature is that, today, much of the responsibility for promoting PA among disabled people lies at the feet of the latter group (Kime et al., 2020). While some HCPs consider PA to be beyond their expertise and remit, and less important than other health promotion activities (Albert et al., 2020; Din et al., 2015; Glowacki et al., 2019), embedding PA promotion in healthcare has been established as a strategic priority supported by the evidence (Gates & Ritchie, 2018; Kime et al., 2020; Milton et al., 2020; Vishnubala & Pringle, 2021). As one example, Sport England indicated that 'if one in four of the inactive population received and acted on advice from their healthcare professional there would 2.9 million less inactive adults in England'. Following from this, a number of national programmes such as 'Making Every Contact Count', 'Movement for Movement' and 'Moving Health Care Professionals' are making important investments in education and training matters. Thousands of HCP are now being trained to include PA conversation in their practice.

Although it remains vital to train the HCPs of today and tomorrow, concentrating all efforts and resources in this professional group is problematic for (at least) two reasons. First, key stakeholders and HCPs themselves highlighted the increasing demands placed on them following global pandemics and expressed the belief that they should not be the only workforce being trained in PA promotion (Netherway et al., 2021). Second, HCPs are not always as relevant to disabled people (when it comes to PA messaging) as the previous literature and national health services often assume. This was made evident in a recent UK‐based study co‐produced with hundreds of disabled people (Smith & Wightman, 2021). These people said that there is another messenger group that has been overlooked, but that would be better than HCPs, even preferable to community‐based organisations. That is social workers. To be clear, their point was not that social workers need to substitute the other messenger groups and make them obsolete. Rather, disabled people identified social workers as a professional force that can increase their chances to become, and stay, physically active. The rationale why disabled people identified social workers as people they want to deliver PA is diverse and compelling. Such rationale is detailed in Smith and Wightman (2021) and therefore does not require to be fully repeated here. Suffice to say that disabled people insisted that social workers have a great reach, are credible, empathetic, concerned about their wellbeing, and knowledgeable about their needs and rights. In view of this, Smith and Wightman (2021) asked over 100 social workers about the possibility of becoming PA messengers. While stressing workload constraints, their response was affirmative. Currently, however, this opportunity for PA promotion cannot materialise, as there is no formal training and education for social workers to learn about how to offer appropriate PA guidance and become confident and consistent messengers. 'Moving Social Work' is the first attempt to change the widespread lack of training and education in this workforce.

**FIGURE 1:**
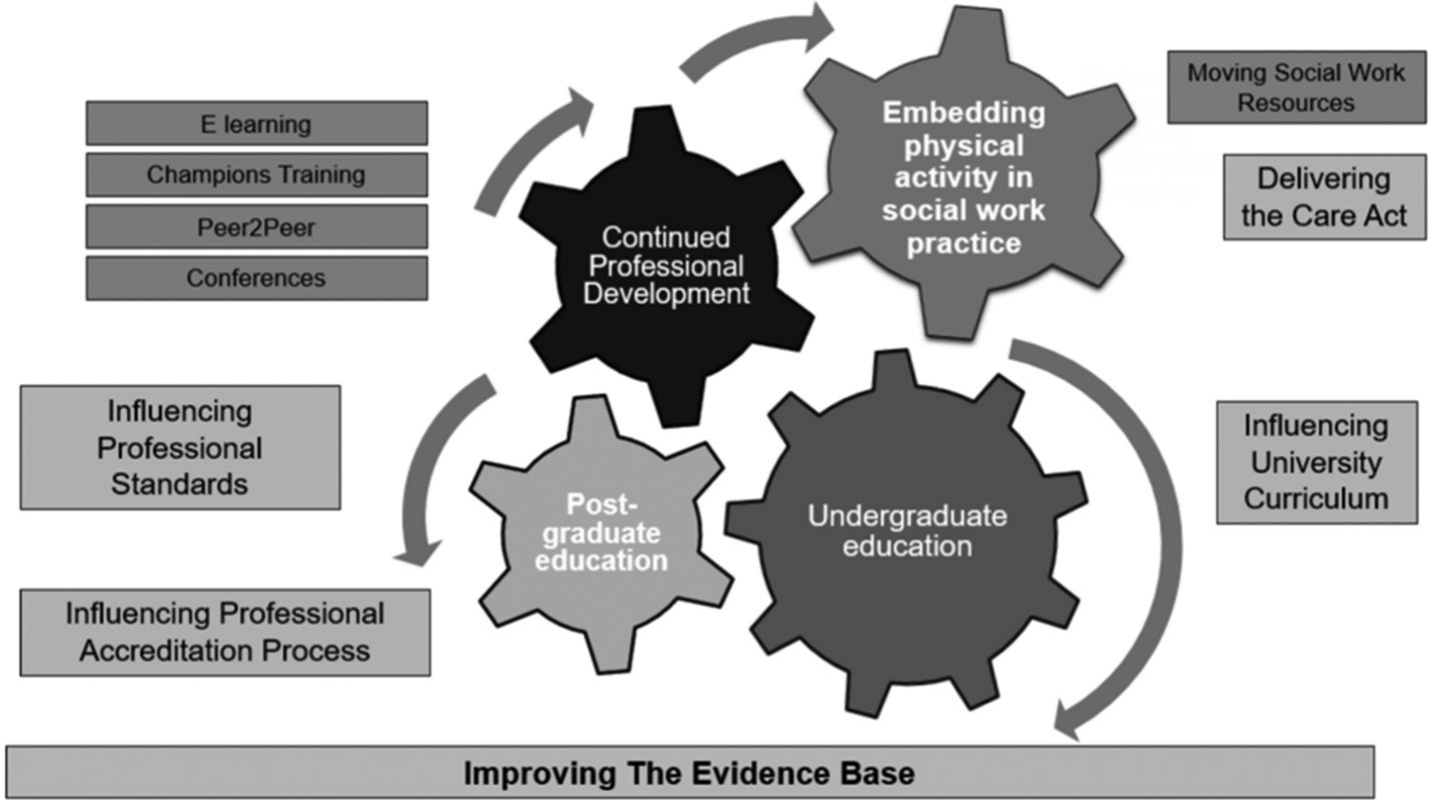
The Moving Social Work programme diagram

'Moving Social Work' is a research project funded by the National Institute of Health Research (NIHR) and Sport England, as well as endorsed and supported by Social Work England, Disability Rights UK, and the Office for Health Improvement and Disparities, amongst other stakeholder organisations. The core plan of the project is to develop a training and education programme that will spiral through a career of a social worker in order to ensure PA guidance is embedded in the culture of social work, is cost‐ effective, and provides long‐term, sustainable change (Figure 1). For this, a necessary first step is to design a programme prototype. The present study is conducted in the context of such initial step; its overarching purpose is to inform the design of the prototype programme that will then be discussed and refined with community members (e.g., disabled people, social workers, university lecturers) and, next piloted and evaluated in university courses and continued professional development.

Prior to this present study, we reviewed the specialised literature on how HCPs in the UK are trained in PA promotion, as the extant academic literature on medical curriculum design can be a useful context to learn from (Netherway et al., 2021). However, obtaining a more comprehensive and context‐relevant view necessitates additional perspectives that can provide valuable input into the core elements of the future training programme. Therefore, this study takes advantage of the Delphi methodology, a consensus‐building methodology to researching the opinions of qualified experts from diverse backgrounds. Over the last years, the Delphi process has been conceptually and methodologically developed (e.g., Chalmers & Armour, 2019; Hirschhorn, 2019), and several studies have employed it successfully to inform core curriculum design (e.g., Brujins et al., 2020; Clayton et al., 2006; Fallon & Trevitt, 2006; Wattanapisit et al., 2019). Along the line of these studies, our article aims to reach expert consensus to answer the following research questions:

**RQ1:** What are the essential contents that social workers need to learn throughout the training programme?
**RQ2:** What are the most appropriate teaching methods to deliver such contents?
**RQ3:** What are the key factors that can jeopardise the success of the training programme?

## 2 METHOD

### 2.1 The Delphi design

Conducting a Delphi study involves distributing a sequence of tailored questionnaires to a sample of selected experts until consensus among them is reached, or until their opinions are stable across questionnaire rounds (Hirschhorn, 2019). Expert selection is intentional or subjective, and the sample size is not attempted to be statistically representative. Instead, representativeness is ascertained through the quality of the panel (Sinclair et al., 2016). The definition of consensus in Delphi studies is a contentious issue. Still, the most utilised and practical definition for expert consensus is percent agreement, with some studies setting the level of agreement as low as 50% and others as high as 100% (Powell, 2003).

According to Chalmers and Armour (2019), the Delphi design is grounded on three core features: '(1) anonymous group interactions and responses, (2) multiple rounds of questioning, and (3) the provision of feedback to the group between each round' (p. 41). Such features are meant to protect the identity of the participants, stimulate reflection, facilitate the convergence of opinions and prevent both the potential effect of dominant figures and conflict among peers. Beyond the above tenets, the Design of a Delphi is flexible. As Hirschhorn (2019) clarified, this means that the researchers can customise the process based on the singularities and contingencies of the research context.

At present, especially in the age of Covid‐19, Delphi studies are usually executed online. One benefit of the online Delphi for the researchers is the possibility to reach experts from different geographical locations. Meanwhile, a key advantage for the experts is the possibility to complete the questionnaires at a time of their comfort. Like other Delphi studies relating to health care and curriculum development (e.g., Salmon & Thombs, 2018), our study was developed using a secure and effective survey tool, namely, Bristol Online Survey. We invited experts to participate in three questionnaire rounds. Using open‐ended questions, round 1 was designed to collect expert opinions; round 2 involved scoring the opinions; finally, round 3 entailed reappraising the scores and generating a final summary of the opinions.

### 2.2 Expert selection and recruitment

As Okoli and Pawlowski (2004) highlighted, building an appropriate expert panel is the most important part of any Delphi study. The panel for this study comprised experts in one or more of these three domains: social work, disability, and physical activity and health. With the co‐production group that is comprised of disabled people, social workers, university social work lecturers and physical activity professionals working with disabled people in the community, we established different expert categories including government authorities, academics with background knowledge in pedagogy and teaching, relevant practitioners, team managers and directors of non‐profit organisations. Likewise, participants had to match at least one of the following conditions: affiliation to eminent organisations or universities, decision‐making roles in key charities or services, or involvement in government entities, including national entities and councils. Some participants identified worked across domains and expert categories. By way of example, one participant of the study is an expert of disability and sport, but also conducts academic work and collaborates with diverse organisations aiming to support social workers in England. Finally, it was established that the sample needed to include disabled experts, experts who also act as carers of disabled people and experts who work closely for and with disabled people. Consistent with the Delphi design, a purposive method of sampling was used to ensure 'that particular categories of cases within a sampling universe are represented in the final sample' (Robinson, 2014, p. 32). Several contacts were obtained through some of the authors' previous connections. For instance, several qualified experts were identified from the connections that the third author established over the last years while leading the 'Get Yourself Active' project (Smith & Wightman, 2021) and the co‐production of the UK Chief Medical Officers’ physical activity guidelines (Smith et al., 2018). Along with co‐production group, stakeholder groups supporting 'Moving Social Work' (e.g., Disability Rights UK) and members of the advisory board of the project (including disabled people) contributed to identify and contact key influencers. Finally, the snowball sampling technique was used to recruit so‐called 'hard‐to‐reach' experts. Following the nomination process, 60 people were approached and invited to participate in the study. We provided them with a project summary and offered them a £20 gift card based on them the completing all the questionnaire rounds. Most experts expressed their interest in the study. Before starting the data collection process, we received the approval of the ethics committee of Durham University (SPORT‐2020‐02‐18T17_18_37‐dmgf98).

## 3 DATA COLLECTION AND ANALYSIS

### 3.1 Questionnaire round 1: Brainstorming

At the end of April 2021, the experts were sent an email with a link to the initial questionnaire, a privacy note and further information about the research. They were also invited to visit a blog of the study, which was created following Hirschhorn's (2019) suggestion of establishing a separate communication channel for those interested in learning more about the questionnaire's motivation and aims. The basic aim of the questionnaire was to identify items around each of the three research questions. After providing informed consent, experts were asked to list up to seven items, alongside a short description and a brief rationale for including each of the items. The following problems were presented to the participants:

**RQ1:** The training programme will include a set of contents that social workers will have to learn to successfully promote physical activity among disabled people. Please list up to seven essential contents that should be taught in the programme. If possible, explain very briefly why you have chosen each of the seven contents. Examples of contents that you can list are: The definition and types of PA; the FITT principle (frequency, intensity, time and type); and the benefits of PA. You can include such items in your response.
**RQ2:** It is important to deliver the training contents through effective teaching methods. Please list up to seven teaching and learning methods that could be used to train social workers on how to promote physical activity among disabled people. If possible, explain very briefly why you have chosen each of the seven teaching methods. Examples of teaching and learning methods that you can list are: interactive activities; tutorials and project‐based learning. You can include such items in your response.
**RQ3:** Different barriers can hinder the success of the training programme. It is important to anticipate these potential barriers from the programme design stage. Please list up to seven potential barriers to implementation of physical activity promotion training in social work education. If possible, explain very briefly why you have chosen each of the seven barriers. Examples of perceived barriers to success that you can list are: curriculum overload; lack of interest and tensions with professional identity. You can include such items in your response.

The three examples provided in each of the problems were selected according to previous literature. For example, the definition and types of PA; the FITT principle (frequency, intensity, time and type); and the benefits of PA are contents that experts participating in the Delphi study of Wattanapisit et al. (2019) considered essential within training programmes in PA promotion for medical curricula.

Responses arrived in sporadic amounts. Reminder emails were sent after a week to those who had not responded, with two further reminders sent before the survey closed (1 month after opening). In many cases, we received automatic responses indicating the participants' unavailability. In a Delphi, as Hirschhorn, Veeneman and van de Velde (2018) pointed out, 'the survey's coordinator has no ability to enforce participation of invited experts and having a low turn‐out is a significant risk' (p. 146; see e.g., Boardley et al., 2021). This is especially so in the case of prominent experts, who have very limited time availability and can receive hundreds of e‐mails every day. The pressures exerted on people during Covid‐19 may have as well added to experts’ ability to engage in research. Eventually, 33 of the 60 identified experts completed the round one online questionnaire (55% response rate).

Answers to open‐ended questions were coded by the first author through a content analysis. Coding served to identify patterns, remove redundancies and generate three inventories: the content list; the teaching method list and the barriers to success list. Despite the appeal and wide use of inter‐rater reliability and member checking as methods to keep coding reliable, such methods were not used due to major problems detailed in Smith and McGannon (2018). Such problems do not apply to quantitative work but appear when a qualitative logic is adopted. From this logic, there is no evidence base to support member checking and inter‐rater reliability as verification methods. Indeed, it is suggested that reliability is not an appropriate criterion to control the quality of qualitative procedures such as coding (Smith & McGannon, 2018). Given so, the lists resulting from the analysis were developed and refined through other methods, these being member reflections and critical friends. This move might be seen as a modest innovation within Delphi studies, whose emphasis on demonstrable agreement may have hidden possibilities beyond reliability testing. Member reflections are akin to member checks, but they are not employed to 'verify' the results. Rather, they serve the purpose of exploring 'any gaps in the results or similarities they share concerning interpretations of the findings' (p. 108). In the same vein, critical friends are not used to 'find correspondence with the truth', but to obtain feedback that illuminates possible interpretations and framings (Smith & McGannon, 2018). Within our coding process, critical friends included the second and third author, members of the co‐production group, and the advisory board members of the project. Building on the received feedback, the first author generated the final output of this round.

### 3.2 Questionnaire round 2: Scoring items

In the second questionnaire, experts were asked to rate the items of each list according to their perceived importance. For that, we established the following scoring frame: 1 = Indispensable; 2 = important; 3 = Moderately important; 4 = Optional; 5 = Unnecessary. Experts could only select one score per item, and they were required to score all items. Twenty‐six experts completed the second questionnaire (79% response rate) during the month of June. The mean score, the standard deviation and mode for each item rated by experts were calculated. The data analysed were presented to them in an easy‐read format. They were then invited to consider their individual responses and the collective results of the round with an eye to the next round.

### 3.3 Questionnaire round 3: Producing a summary

Within the month of July 2021, 20 experts responded to the final questionnaire round (77% response rate), either modifying or ratifying their previous scores. Table 1 presents the basic characteristics of the panel members that completed the three questionnaire rounds. Considering that the minimum number of experts in a Delphi study is 5 (Clayton et al., 2006), 20 represents an appropriate panel size, similar to relevant Delphi Studies in curriculum development and public health (e.g., Guan et al., 2019; Moynihan et al., 2015; Salmon & Thombs, 2018; Wattanapisit et al., 2019). Consensus was determined by having 80% of participants' scores within two categories—important (score = 2) and indispensable (score = 1). In other words, achieving consensus meant that the item was deemed important or indispensable by at least 80% of experts. This consensus rule has been used by authors such as Sinclair et al. (2016) and Stewart et al. (2017). When some items do not reach the established consensus threshold this does not necessarily mean that such items are unsuitable ingredients for the training of social workers. As Powell (2003) reminded, consensus in a Delphi represents expert judgment rather than indisputable fact, and it is sensitive to reader's interpretation. More will be said about the meaning of consensus in the discussion section.

**TABLE 1:** Characteristics of the panelists

	*n* (%)		
	Round 1	Round 2	Round 3
Sex^a^			
Male	15(45)	13(50)	9(45)
Female	18(55)	13(50)	11(55)
Main field of expertise			
PA & Health	12(36)	10(38)	8(40)
Disability	12(36)	9(35)	6(30)
Social Work	9(27)	7(27)	6(30)
Years of experience			
1–5	3 (9)	1(4)	1(5)
5–10	5(15)	4(15)	4(20)
10–20	13(40)	11(42)	8(40)
>20	12(36)	10(39)	7(35)

^a^ Experts were given the option to select ‘Non‐binary'and "Prefer to not declare".

## 4 RESULTS

### 4.1 Contents

**FIGURE 2:**
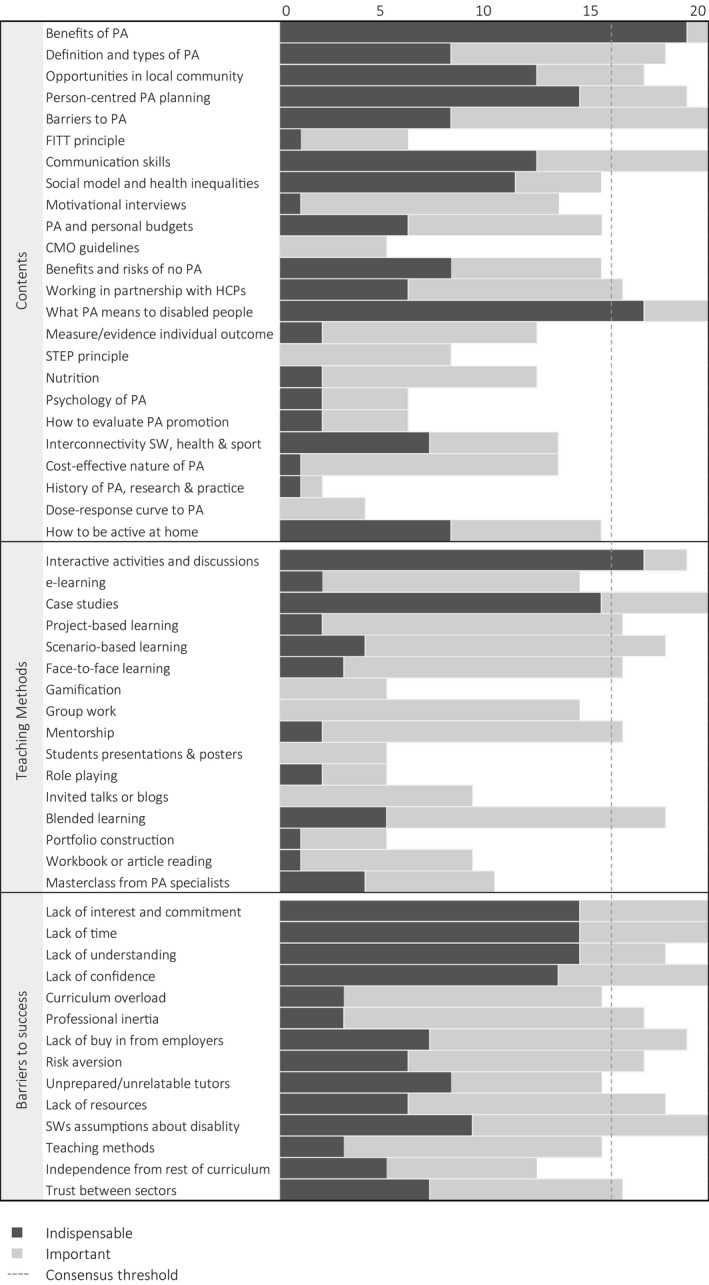
The number of experts in round three (n= 20) who considered the listed items indispensable or important

The content list generated from round 1 was composed by 23 items. Table 2 presents the most frequently proposed items, together with a simplified summary of the experts' descriptions and arguments. Table 3 presents the results of the two scoring rounds. Here, it can be observed the general stability of the ratings. In round 3, the expert panel reached consensus on eight contents. Four of them achieved an agreement of 100%, namely: communication skills, barriers to PA, benefits of PA and what PA means to disabled people. The last two contents of this list were deemed to be 'indispensable' by >80% experts. The item 'social model of disability and health inequalities' did not achieve consensus but was marked as 'indispensable' by 55% of the experts. As observable in Figure 2, no other item that did not reach consensus was deemed 'indispensable' by as much as half of the panel. Therefore, this content might be also considered worth teaching.

**TABLE 2:** The most frequently proposed contents resulting from analysing the participants' responses in Round 1

Item	Descriptions provided by the experts	Reasons provided by the experts	Number of experts
Benefits of PA	Physical and psychosocial benefits of doing PA, with a particular focus on benefits to people experiencing different impairments and people who are less active.	To show Social Workers why PA is so important to overall health and wellbeing and needs to be prioritised.	23
Definition and types of PA	The range of physical activities that can be promoted among disabled people.	To increase knowledge and awareness—not everyone knows what is available.	19
How to find opportunities in the person's local community	How to find the right services/agencies/resources that will help identify accessible activities in the person's local community.	To know where the best opportunities are and their costs.	15
Person‐centred PA planning	How to identify appropriate and realistic PA for each person, and how to include PA in care plan reviews.	To emphasise that there is not a 'one size fits all' approach, ensure PA can be personally enjoyable, and promote *with* and not just *for* disabled people.	15
Barriers to PA	Intrapersonal, interpersonal, organisational and communitary factors that hinder PA participation. Not only identify but use examples and learn how to address them.	Disabled people remain inactive partly due to multiple barriers to PA.	12
FITT principle	Frequency, Intensity, Time and Type.	To help disabled people understand how long and how hard they should exercise.	8
Communication skills	Skills for communicating PA messages with disabled people successfully. (Emphasis should be put on listening skills).	Needed for effective promotion of physical activity.	8

**TABLE 3:** The most frequently proposed teaching methods resulting from analysing the participants' responses in Round 1

Item	Descriptions provided by the experts	Reasons provided by the experts	Number of experts
Interactive activities and discussions	Discussion‐based learning. Presenting problems and situations to stimulate debate, reflection and learning.	It gets people thinking and involved, and it adds value in terms of discussion and learning from others' practice and knowledge.	14
e‐Learning	Online teaching and resources, including webinars.	It enables people from many different locations easy access to learning with the inclusion of break out rooms for discussion and questioning. It is quick and efficient and allows social workers work at their own pace.	13
Case studies	For example, the case of a disabled person who has become more active with the support of their social worker, and the difference this has made to them. (Other kinds of cases are possible). One possible format to display the cases is video.	To demonstrate the impact (not just to physical health but social connections, wider family impact, sometimes even training and employment).	11
Project‐based learning	Students gain knowledge and skills by working to respond to a question, problem, or challenge.	By working on a project, students will most likely retain and develop skills, confidence, and commitment to supporting disabled people to access and enjoy PA.	10
Scenario‐based learning	Give students the chance to join in disabled sports activities visiting a fitness/leisure site.	It allows social workers observing how things work in the community.	8
Face‐to‐face learning	Contents are thought in person.	This method is challenging in terms of Covid risk, but it offers an appropriate learning environment, feedback, and interactions.	7

### 4.2 Teaching methods

In round 1, the experts referred to 15 teaching methods that could be used to deliver the contents proposed above. A sample of the most popular teaching methods mentioned by the experts is given in Table 4. All the experts who participated in the last round agreed that case studies should be used for training social workers in PA promotion. However, interactive activities and discussions were the only item considered ‘indispensable’ by more than 80% of the experts (Table 5). Following round 3, 7 teaching methods crossed the consensus threshold (Figure 2). It is noteworthy that face‐to‐face learning (80%) and blended learning (90%) reached consensus, but not so e‐learning (70%). This suggests, first, that the former could be used alone, but not so the latter; and second, that blended learning is a better option as it can contain both in person and e‐learning.

**TABLE 4:** The most frequently proposed challenges resulting from analysing the participants' responses in Round 1

Item	Descriptions provided by the experts	Reasons provided by the experts	Number of experts
Lack of interest and commitment	PA might not interest some students/social workers, or not seen as priority, or not seen desirable (given past negative experiences). It is necessary to promote the idea of promoting PA before offering the training.	Students may switch off from the beginning.	22
Lack of time	Social workers are overworked and do not have time to learn PA promotion.	No arguments provided.	17
Lack of understanding	Lack of understanding of the importance of PA and how this would benefit service users.	It prevents engagement and can impact on lack of interest.	14
Lack of confidence	Social workers may not feel confident enough to learn PA promotion and deliver PA messages, specially if lifestyle choices contradict the message giving	No arguments provided.	9
Curriculum overload	Lack of space in the curriculum for PA contents	No arguments provided.	7
Professional inertia	PA is not in role. It is not relevant to social work standards, it is perceived to fall outside the conventional Social Worker's core role	Social workers' practice does not use PA interventions currently, therefore will not be promoted as a tool for social workers in practice	6
Lack of buy in from employers	Employers/senior managers might not accept the new role of social workers. This new role can have a poor impact in social worker's careers, and they might have little incentives to promote PA	The PA training can have no real influence on social worker's future role/career, and they might ask themselves if it is worthy.	5

**TABLE 5:** Contents

Items (*n* = 24)	Round 2 agreement^a^, mean ± *SD*	Round 3 agreement^a^, mean ± *SD*
Benefits of PA^b^	100%, 1.27 ± 0.44	100%, 1.05 ± 0.22
Definition and types of PA	88%, 1.65 ± 0.68	90%, 1.70 ± 0.64
Opportunities in the person's local community^b^	88%, 1.69 ± 0.77	85%, 1.60 ± 0.86
Person‐centred PA planning^b^	92%, 1.42 ± 0.63	95%, 1.35 ± 0.57
Barriers to PA	81%, 1.85 ± 0.82	100%, 1.60 ± 0.49
FITT principle	46%, 2.85 ± 1.17	30%, 3.05 ± 0.97
Communication skills^b^	96%, 1.54 ± 0.57	100%, 1.40 ± 0.49
Social model of disability and health inequalities^b^	58%, 2.15 ± 0.99	75%, 1.75 ± 0.94
Motivational Interviews	58%, 2.19 ± 0.96	65%, 2.35 ± 0.65
PA and personal budgets	65%, 2.23 ± 0.97	75%, 2.15 ± 1.11
CMO guidelines	31%, 3.00 ± 0.92	25%, 3.05 ± 0.80
Benefits and risks of no PA^b^	50%, 2.38 ± 0.88	75%, 2.00 ± 1.10
Working in partnership with healthcare professionals	77%, 2.00 ± 0.68	80%, 1.90 ± 0.70
What PA means to disabled people^b^	81%, 1.81 ± 0.83	100%, 1.15 ± 0.36
Measure/evidence individual outcomes	58%, 2.54 ± 0.93	60%, 2.45 ± 0.92
STEP principles	42%, 2.88 ± 1.09	40%, 2.90 ± 0.89
Nutrition	46%, 2.88 ± 1.01	60%, 2.50 ± 0.97
Psychology of PA	35%, 3.00 ± 1.07	30%, 3.10 ± 1.09
How to evaluate PA promotion	31%, 3.08 ± 1.03	30%, 2.95 ± 1.07
Interconnectivity between social work, health and sport^b^	58%, 2.27 ± 1.16	65%, 2.15 ± 1.06
Cost‐effective nature of PA	46%, 2.69 ± 0.99	65%, 2.50 ± 0.92
A short history of PA, research, and practice	08%, 3.69 ± 0.87	10%, 3.65 ± 0.96
Dose–response curve to PA	15%, 3.50 ± 0.89	20%, 3.45 ± 0.97
How to be active at home^b^	58%, 2.19 ± 0.88	75%, 1.90 ± 0.89

^a^ Percentage of experts who scored 1 and 2 for each item.

^b^ Items achieved a mode of ‘Indispensable’ in the final round

### 4.3 Barriers to success

Experts proposed 14 challenges or barriers that the training programme will need to address in order to be successful (Table 6). Except 4, all items reached consensus (Table 7). For the experts, it was not so much the presence but the absence of some factors that can present problems for the effectual development of the training programme. Specifically, experts' concerns were directed mainly towards the mindset of social workers: their lack of interest and commitment, understanding, and confidence, for example. Their assumptions about disabled peoples' capacities, their fear to recommend inappropriate PA information, and their perception that PA messaging is not part of their profession were also seen as potential hindering factors. Interpersonal issues such as the attitude of their employers and members of other sectors (e.g., healthcare) towards PA promotion can be important obstacles as well. Finally, organisational barriers such as curriculum overload and lack of resources were deemed potential stumbling blocks to consider.

**TABLE 6:** Teaching methods

Items (*n* = 16)	Round 2 agreement^a^, mean ± *SD*	Round 3 agreement^a^, mean ± *SD*
Interactive activities and discussions^b^	100%, 1.31 ± 0.46	95%, 1.20 ± 0.51
e‐Learning	73%, 2.12 ± 0.93	70%, 2.20 ± 0.60
Case studies^b^	73%, 1.73 ± 1.02	100%, 1.25 ± 0.43
Project‐based learning	81%, 2.00 ± 0.73	80%, 2.15 ± 0.65
Scenario‐based learning	77%, 1.92 ± 0.73	90%, 1.90 ± 0.54
Face‐to‐face learning	81%, 2.04 ± 0.81	80%, 2.10 ± 0.70
Gamification	19%, 3.31 ± 1.10	25%, 3.25 ± 0.99
Group work	58%, 2.42 ± 0.93	70%, 2.40 ± 0.66
Mentorship	54%, 2.42 ± 0.88	80%, 2.20 ± 0.75
Student presentations and posters	31%, 3.19 ± 1.00	25%, 3.10 ± 0.77
Role playing	23%,3.38 ± 0.88	25%, 3.20 ± 1.08
Invited talks or blogs	39%, 2.85 ± 1.03	45%, 2.80 ± 0.87
Blended learning	65%, 2.15 ± 0.82	90%, 1.85 ± 0.57
Portfolio construction	31%, 3.23 ± 1.05	25%, 3.40 ± 1.07
Workbook or articles reading	27%, 3.12 ± 0.93	45%, 2.95 ± 1.12
Masterclasses from different types of PA specialists	50%, 2.50 ± 1.12	50%, 2.50 ± 1.07

^a^ Percentage of experts who scored 1 and 2 for each item.

^b^ Items achieved a mode of 'Indispensable' in the final round.

**TABLE 7:** Barriers to success

Items (*n* = 14)	Round 2 agreement^a^, mean ± *SD*	Round 3 agreement^a^, mean ± *SD*
Lack of interest and commitment^b^	96%, 1.31 ± 0.54	100%, 1.30 ± 0.46
Lack of time^b^	88%, 1.62 ± 0.79	100%, 1.30 ± 0.46
Lack of understanding^b^	85%, 1.65 ± 0.73	85%, 1.50 ± 0.74
Lack of confidence^b^	89%, 1.54 ± 0.69	95%,1.45 ± 0.59
Curriculum overload	58%, 2.35 ± 0.96	85%, 2.15 ± 0.73
Professional inertia	65%, 2.23 ± 1.12	85%, 2.05 ± 0.67
Lack of buy in from employers	73%, 2.00 ± 0.83	95%, 1.75 ± 0.70
Risk aversion	73%, 1.96 ± 0.76	85%, 1.85 ± 0.65
Unprepared or unrelatable tutors/trainers^b^	62%, 2.19 ± 1.04	75%, 1.85 ± 0.79
Lack of resources	66%, 2.19 ± 0.96	80%, 1.85 ± 0.73
Social workers' assumptions about disability	81%, 1.88 ± 0.70	95%, 1.60 ± 0.58
Teaching methods	61%, 2.27 ± 1.06	75%, 2.10 ± 0.62
Independence from the rest of social work curriculum	46%, 2.65 ± 1.14	60%, 2.35 ± 1.11
Trust between sectors	61%, 2.42 ± 1.08	80%, 2.00 ± 1.05

^a^ Percentage of experts who scored 1 and 2 for each item.

^b^ Items achieved a mode of ‘Indispensable’ in the final round.

## 5 DISCUSSION, KEY MESSAGES, AND FUTURE STEPS

In the UK, programmes such as 'Moving Healthcare Professionals' provide training for HCPs, equipping them to promote PA among their patients. 'Moving Social Work' offers something similar, with two major differences. First, it provides training to social care professionals, specifically social workers; and second, it focusses on the promotion of PA for disabled people. ‘Moving Social Work’ can and should learn from the literature and models capturing healthcare education and training (Netherway et al., 2021). However, we cannot merely imitate the HCP curriculum without considering contextual differences. That is why we have gathered the opinions of experts on PA and health, social work and disability, in order to reassess our observations from the available literature and inform the design of a specialised training programme prototype for social workers. The results of this Delphi study confirm that, while the HCP curriculum is a valuable referent, a training programme for social workers asks for something slightly different, it brings something different to the table, and presents unique challenges. What are the prominent points that this study brings to the table?

First, while health care providers are generally aware of the importance PA and believe that they have a role in promoting PA as part of the clinical practice (Hébert et al., 2012; Pugh et al., 2020; Williams et al., 2018), experts in this study suggested that social workers might not recognise the value of promoting PA as part of their job. This means that addressing limited time, confidence and access to appropriate resources might not be enough. To create genuine impact, the idea of promoting PA needs to be actively promoted amongst those social workers who perceive there to be little or no meaning in this new role (i.e., PA messengers). As the experts in this study equally proposed, we also need to show employers, senior managers and members of other sectors that preparing social workers for PA messaging is an initiative worth pursuing. Doing this effectively is an added challenge that the 'Moving Social Work' project is required to consider.

Second, the benefits of PA and the definition and types of PA appear to be important both for HCP and social workers, but other elements that are central in the healthcare curriculum, such as the FITT principle and the CMO guidelines, are not considered relevant for the social work curriculum. Simultaneously, contents such as the meaning of PA and barriers to PA are deemed necessary here but are rarely considered in the HCP curriculum. We see these differences as convenient. For one, it is more likely that social workers become enthusiastic about learning how to include PA in disabled individuals’ care plan, compared with learning epidemiological content such as the dose–response curve to PA. For another thing, social workers and HCP need different skills, and thus these differences can complement each other. Here, it is vital ‐as the experts in this study indicated‐ that both workforces learn to work in equal partnership. This means that, in the future, HCPs education will need to include content on how to collaborate with social workers to make every contact count for PA.

Third, this study suggests that some teaching methods might be more fitting than others. Experts in this study considered blended learning to be more appropriate than e‐learning *or* face‐to‐face learning, even though the latter obtained consensus. For HCPs, blended learning is relatively new, but has shown promising results, especially when students and teachers exchange views and interact with each other (Westerlaken et al., 2019). In this study, consensus on the value of interactive activities and discussions was particularly strong. This result indicates, albeit indirectly, that experts value the co‐creation of knowledge and meaning and reflexive thinking. Other nominated methods such as scenario‐based learning, case studies and project‐based learning are also aligned with developing these capacities. This research did not address learning evaluation and assessment, but the results invite us to think that continuous assessment (as opposed to the final examination model) will be a coherent approach. Even so, this issue will be examined in future research.

The essential contents and teaching methods agreed by the experts can be creatively assembled in teaching units. For example, a teaching unit can be developed around the case of Alison, a person with a spinal cord injury. Alison can be either a fictional character or a real person. The case can describe what does PA means to Alison, and which are the key benefits according to her impairment characteristics and personal interests. Using a given scenario as a prompt, students can be asked to provide Alison with personalised guidance on how to be active her way within her local community, considering the barriers that she might experience, such as lack of transport and neuropathic pain. Next, students can be provided with diverse resources and information, then asked to work together to select the most suitable to pass to Alison using effective communication strategies. In this example, some of the contents and teaching methods that experts put emphasis on are put together to create a pedagogical whole. Different combinations are possible.

Despite such possibilities, this study cannot deliver a ready‐to‐use training programme. One reason for this is that Delphi studies are grounded on the assumption that uniformity of belief and evaluation are the standard against which to make decisions. While conflict is not completely ignored, the Delphi risks treating conflict as a mere residual category; it prioritises 'a thin agreement at the lowest common denominator' (Hillier, 2003, p. 43) at the expense of the rich meaning that conflicting differences between groups and individuals can generate. To address this shortcoming, we will conduct follow‐up interviews with at least 10 of experts that participated in all the questionnaire rounds, using the results of the Delphi as prompts to stimulate dialogue and gain understandings that go beyond identification and prioritisation. This interview‐based study was not originally included in the project design; the necessity of conducting it emerged from discussion with the co‐production group. Following further discussion, it has been decided that the interviewers will comprise an academic and two members from the group, while the rest of members will keep involved as critical friends, influencing the research agenda and process. In addition to this follow‐up study, other approaches such as the 'Knowledge Café' (see e.g., Löhr et al., 2020) will be used to gather more knowledge from experts by experience, including social work students, educators and practitioners, disabled people, representatives from disabled people's user‐led organisations, and people working in local communities who promote PA.

Although this Delphi study serves the purposes of the 'Moving Social Work' project, it can also be useful outside its boundaries. For example, the results of the study can raise awareness about the potential impact derived from training social workers in the UK, but also in other countries. Equally, the study can open the possibility for considering social workers in future PA promotion for other groups suffering from health and social inequalities, such as refugees or sex workers. In pursuing these potential directions, it will be useful to consider organisational, cultural and community factors, as opposed to assuming that work from one country around one population group can be merely transported into other countries and groups. Another example of how academics and non‐academics can benefit from this Delphi study has to do with its potential for provocative generalisation. For Fine et al. (2008), this form of generalisability refers to research that provokes readers to rethink 'the possible' and asks researchers to 'move their findings towards that which is not yet imagined, not yet in practice, not yet in sight' (p. 169). On the one hand, the results of this study might provoke both the social care and the healthcare sector to recognise social workers as PA messengers, or to change their perception about what and how PA promotion is learned. On the other hand, the results might elicit some critical reactions from disabled activists, for instance, which perhaps could use the study to draw the attention to an overlooked but crucial content worth teaching. As Smith (2018) would suggest, it is now on the readers to engage with the study, and then either support or reject its results as provocatively generalisable to them.

## 6 CONCLUSION

This article has provided a partial but strong base to design the first training programme prototype that will prepare social workers to effectively promote PA to and for disabled people in the UK. Using a Delphi method, we have identified and prioritised training components, as well as challenges and gaps that will be addressed in the next stages of the programme design. We invite readers to look at this study as part of a broader research which requires time to generate impact. In particular, we invite you to follow ‘Moving Social Work’ through the communication channels of Sport England, Disability Rights UK and the NIHR, as well as the Twitter account of the project (@MovingSW).

## CONFLICT OF INTEREST

The authors declare that they have no conflict of interest.

## AUTHOR CONTRIBUTIONS

All authors contributed to the manuscript substantially and approved the final version.

## Data Availability

The data that support the findings of this study are available on re‐quest from the corresponding author. The data are not publicly avail‐able due to privacy or ethical restrictions.
